# Durable complete response to tislelizumab in advanced pMMR cervical cancer and successful rechallenge after immune-related NSTEMI: a case report

**DOI:** 10.3389/fphar.2026.1947402

**Published:** 2026-10-05

**Authors:** Qingyun Zhang, Xiaoli Wang, Ying Chen, Hui Feng, Xingning Fu, Haipeng Ren, Xin Wu

**Affiliations:** 1 Department of Oncology, Weifang People’s Hospital (The First Affiliated Hospital of Shandong Second Medical University), Weifang, China; 2 Department of Breast and Thyroid Surgery, Sunshine Union Hospital, Weifang, China; 3 Department of Pathology, Sunshine Union Hospital, Weifang, China; 4 Center for Precision Pathological Diagnosis, Weifang People’s Hospital (The First Affiliated Hospital of Shandong Second Medical University), Weifang, China

**Keywords:** accelerated atherosclerosis, advanced cervical cancer, immune checkpoint inhibitors, immune-related adverse events, non-ST-segment elevation myocardial infarction, tislelizumab

## Abstract

**Background:**

Immune checkpoint inhibitors (ICIs) have substantially improved long-term oncologic outcomes in patients with advanced malignancies; however, ICI-related cardiovascular adverse events have emerged as a growing clinical challenge. Accumulating clinical evidence confirms that ICI treatment markedly increases the risk of atherosclerotic cardiovascular disease (ASCVD). Although cardiovascular toxicities secondary to ICIs are relatively infrequent, these complications carry severe and potentially fatal clinical consequences. Herein, we describe a rare case of acute non-ST-segment elevation myocardial infarction (NSTEMI) complicated by accelerated atherosclerosis potentially associated with ICI therapy in a middle-aged woman with advanced cervical squamous cell carcinoma harboring proficient mismatch repair (pMMR) status. Following successful coronary revascularization, the patient safely reinitiated immunotherapy and achieved long-term sustained tumor-free survival.

**Case presentation:**

A 39-year-old woman was diagnosed with International Federation of Gynecology and Obstetrics (FIGO) stage IVB cervical squamous cell carcinoma, with comorbid type 2 diabetes mellitus and hypertension. She received first-line quadruple combination therapy comprising nab-paclitaxel, cisplatin, bevacizumab, and tislelizumab. Complete tumor response was achieved following six cycles of systemic treatment. Subsequent maintenance therapy combined with pelvic radiotherapy enabled sustained disease remission for more than 5 years. Approximately 2 years after transitioning to single-agent tislelizumab maintenance, the patient experienced an acute NSTEMI. Coronary angiography demonstrated severe multivessel coronary stenosis, and percutaneous coronary intervention (PCI) was performed successfully. After thorough multidisciplinary cardio-oncology evaluation, tislelizumab was safely reinitiated and continued until August 2024. At the most recent follow-up in June 2026, the patient maintained a persistent complete response with stable cardiac function.

**Conclusion:**

This case highlights that patients with advanced pMMR cervical cancer complicated by diabetes and hypertension can achieve durable complete remission exceeding 5 years with tislelizumab-based combination regimens. Furthermore, this case report also suggests that ICIs may be associated with cardiovascular events secondary to accelerated atherosclerosis, and demonstrates the clinical feasibility of safely resuming immune-checkpoint-inhibitor therapy following myocardial infarction.

## 1 Introduction

The widespread clinical application of immune checkpoint inhibitors (ICIs) has substantially improved long-term survival outcomes in patients with advanced cervical cancer. The KEYNOTE-826 trial demonstrated that pembrolizumab combined with chemotherapy with or without bevacizumab significantly prolongs progression-free and overall survival in patients with programmed death-ligand 1 (PD-L1)-positive advanced cervical cancer, thereby establishing immunotherapy-based combination regimens as the first-line standard of care for recurrent or metastatic cervical cancer (Monk et al., 2023). The cervical cancer clinical practice guidelines issued by the Chinese Society of Clinical Oncology (CSCO) also recommend combination immunotherapy as a core therapeutic strategy (Chinese Society of Clinical Oncology CSCO Guidelines Working Committee, 2025).

Although combination immunotherapy regimens have achieved groundbreaking advances, the vast majority of patients with cervical squamous cell carcinoma harbor proficient mismatch repair/microsatellite-stable (pMMR/MSS) tumors and derive only minimal clinical benefit from single-agent immune checkpoint inhibitor (ICI) therapy. In contrast to dMMR/MSI-H (deficient mismatch repair/microsatellite instability-high) tumors, which display an immune-hot phenotype driven by abundant neoantigen production, pMMR tumors are characterized by a low tumor mutational burden and sparse immune-cell infiltration, collectively conferring a relatively immune-cold phenotype (El-Deiry et al., 2019; Yanaihara et al., 2025). Notably, even among patients with high PD-L1 expression, single-agent ICI therapy fails to confer durable clinical benefit in pMMR tumors. In the KEYNOTE-158 trial, pembrolizumab monotherapy yielded an objective response rate (ORR) of only 14.3% in patients with PD-L1-positive (CPS ≥1) recurrent or metastatic cervical cancer (Chung et al., 2019). Similarly, ORRs of merely 6.5%–14.1% were reported with pembrolizumab monotherapy in patients with pMMR/MSS endometrial carcinoma (O'Malley et al., 2025). Consistent with these findings, multiple clinical trials and meta-analyses have demonstrated that single-agent ICIs produce ORRs generally below 16% in advanced pMMR cervical and endometrial cancers, with complete responses being exceedingly rare (Wang et al., 2024). Accordingly, clinically meaningful and durable anti-tumor responses are seldom observed in patients with advanced pMMR cervical cancer, even in the setting of high PD-L1 expression. Such cases therefore serve as valuable real-world illustrations that underscore the urgent need to develop combination therapeutic strategies for this high-risk advanced cervical cancer population.

Due to the limited efficacy of single-agent immunotherapy, multimodal combination regimens integrating chemotherapy, anti-angiogenic agents, radiotherapy, and ICIs have become a cornerstone strategy for improving immunotherapeutic outcomes in cervical cancer. These four modalities act through distinct yet complementary mechanisms that converge to overcome immune resistance in pMMR tumors. First, chemotherapy reduces tumor burden and induces immunogenic cell death (ICD), releasing tumor-associated antigens that are subsequently cross-presented by dendritic cells to elicit T cell-mediated anti-tumor immune responses (Galluzzi et al., 2024; Janssens et al., 2024). Second, anti-angiogenic therapy normalizes aberrant tumor vasculature by blocking the vascular endothelial growth factor (VEGF) signaling pathway, thereby enhancing the intratumoral infiltration of cytotoxic T cells, curtailing the recruitment of regulatory T cells (Tregs) and myeloid-derived suppressor cells (MDSCs), and ultimately relieving VEGF-mediated immunosuppression (Kabir et al., 2026). Third, radiotherapy induces local tumor cell death by inducing DNA damage and further augments ICD, triggering systemic antitumor immunity termed the abscopal effect. Meanwhile, radiation-driven PD-L1 upregulation promotes adaptive immune resistance, providing a molecular rationale for combining radiotherapy with ICIs (Lu and Deng, 2026). Fourth, ICIs reinvigorate precursor exhausted T cells (TPEX) by targeting the PD-1/PD-L1 axis, facilitating their proliferation and differentiation into functional effector T cells and restoring long-term tumor immune surveillance (Rausch and Kallies, 2025). Collectively, the synergistic interplay among these four therapeutic modalities remodels the immunosuppressive tumor microenvironment into an immune-permissive state, which may underlie the profound and durable tumor regression observed in this patient.

With the expanding clinical use of ICIs, ICI-associated cardiovascular adverse events have become increasingly recognized. Although relatively infrequent, these events can be severe and potentially fatal, underscoring the need for rigorous clinical vigilance and proactive surveillance (Lyon et al., 2018). With the continuous expansion of ICI indications, a wide spectrum of ICI-related cardiovascular toxicities—including myocarditis, accelerated atherosclerosis, acute myocardial infarction (AMI), and ischemic stroke—has attracted growing research interest (Miteva et al., 2025). A meta-analysis incorporating 57 randomized controlled trials with a total of 12,118 participants reported that the overall incidence of ICI-related cardiovascular adverse events reached 8.32% (Xavier et al., 2022). Multiple propensity score-matched cohort studies further confirmed that ICIs markedly increase the risk of atherosclerotic cardiovascular disease (ASCVD); patients receiving ICIs carry a 3.3-fold higher risk than those treated with non-immunotherapeutic regimens (Xavier et al., 2022; Drobni et al., 2020; Gong et al., 2024). Furthermore, the risks of AMI and ischemic stroke are elevated by 51% and 56%, respectively (Palaskas et al., 2024). Notably, patients with cardiovascular and metabolic risk factors including hypertension and type 2 diabetes mellitus face additive cardiovascular hazards (Wang et al., 2025). In routine clinical practice, ICI-triggered accelerated atherosclerosis frequently manifests with atypical symptoms, creating major obstacles to early identification. In addition, no consensus has been reached on the safety and standardized protocols for ICI rechallenge after AMI.

Herein, we report a case of International Federation of Gynecology and Obstetrics (FIGO) stage IVB cervical squamous cell carcinoma with pMMR status, in which the patient achieved a CR on tislelizumab-containing combination treatment, yet developed acute non-ST-segment elevation myocardial infarction (NSTEMI) complicating accelerated atherosclerosis possibly associated with ICI therapy during maintenance immunotherapy. Following percutaneous coronary intervention, the patient safely reinitiated ICI therapy and has maintained durable tumor-free survival to date. This case provides a practical clinical reference for the management of analogous cases.

## 2 Case presentation

A 39-year-old female patient presented with persistent hypogastric pain exacerbated in the afternoon, accompanied by dyschezia, dysuria and scant irregular vaginal bleeding without obvious predisposing causes in May 2020. Initial laboratory testing revealed positive urine ketone bodies, and type 2 diabetes mellitus was tentatively diagnosed. Her abdominal pain was temporarily relieved after symptomatic management. Given recurrent symptoms, she was transferred to the gynecology department in July 2020. The patient had a 3-year history of type 2 diabetes mellitus. Her glycemic status was well controlled with subcutaneous insulin glargine 16 units administered at bedtime, along with oral hypoglycemic medications: linagliptin 5 mg once daily, glimepiride 1 mg twice daily, and pioglitazone 30 mg at bedtime. Glycemic control remained stable during antitumor treatment. She also had a 6-year history of hypertension. Her highest documented systolic blood pressure was 150 mmHg, and her blood pressure measured on admission was 99/68 mmHg. Her long-standing antihypertensive regimen included valsartan 80 mg once daily and sustained-release metoprolol succinate 47.5 mg once daily, with persistently stable blood pressure control. No family history of cardiovascular disease was identified, and the patient denied any history of smoking or alcohol intake. Baseline lipid testing upon admission demonstrated total cholesterol 5.6 mmol/L and triglycerides 1.81 mmol/L, both falling within the institutional reference intervals (total cholesterol: 2.9–6.3 mmol/L). Admission physical examination recorded her height as 157 cm, weight as 62 kg, and body surface area (BSA) 1.59 m^2^, which was calculated using the simplified formula: BSA = (height + weight −60)/100. Pelvic magnetic resonance imaging (MRI) showed abnormal endometrial thickening partially protruding into the cervical canal, along with multiple enlarged pelvic and para-aortic lymph nodes. Hysteroscopic biopsy pathology confirmed moderately to poorly differentiated squamous cell carcinoma involving the endometrium, cervix and vaginal wall.

Positron emission tomography-magnetic resonance (PET-MR) identified a cervical and uterine mass measuring approximately 4.81 cm × 6.8 cm with a maximum standardized uptake value (SUVmax) of 10.61, invading the endometrium and vagina. Widespread metastatic lesions were observed, including bilateral pulmonary metastases, mediastinal and bilateral hilar lymph node metastases, as well as extensive retroperitoneal and peritoneal metastases in the abdominopelvic cavity (Figures 1A,B). Cervicothoracoabdominal computed tomography (CT) further corroborated the above metastatic findings (Figures 2A–D). Clinical stage: FIGO stage IVB cervical squamous cell carcinoma (T3bN1M1, FIGO 2018). Pathological examination of hysteroscopic biopsy tissue: Poorly differentiated carcinoma, favoring a moderately to poorly differentiated squamous cell carcinoma (Figure 1D). Immunohistochemical staining results were as follows: CK5/6 (+), CK8/18 (+), p40 (−) (Figures 1E,F); the combined positive score (CPS) for PD-L1 was 65 (Figure 1G); all mismatch repair proteins including MLH1, MSH2, MSH6 and PMS2 exhibited intact expression (Figures 1H–K), consistent with pMMR status.

**FIGURE 1:**
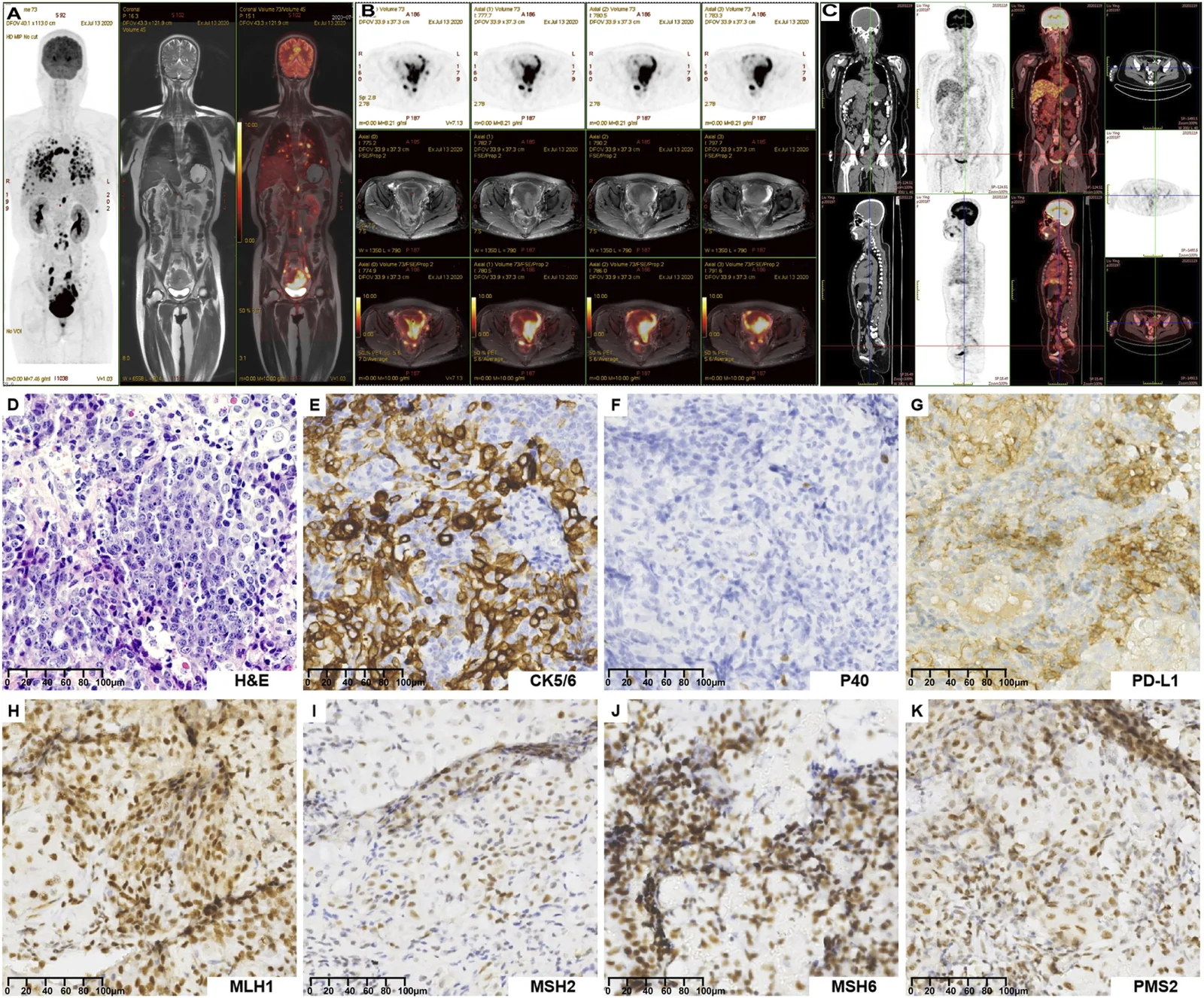
PET-MR and PET-CT evaluation of therapeutic response and pathological characteristics. Baseline PET-MR shows a hypermetabolic cervical-uterine mass (SUVmax = 10.61) with extensive pulmonary, lymph node and abdominopelvic metastases **(A,B)**. Post-induction PET-CT obtained in November 2020 reveals complete resolution of all hypermetabolic lesions **(C)**. Hematoxylin and eosin staining confirms moderately to poorly differentiated squamous cell carcinoma (×20) **(D)**. Immunohistochemistry showed positive CK5/6 and negative p40 (×20) **(E,F)**. High PD-L1 expression with a CPS of 65 (22C3 assay) **(G)**. Intact expression of MLH1, MSH2, MSH6 and PMS2 indicated proficient mismatch repair (pMMR) status (×20) **(H–K)**.

**FIGURE 2:**
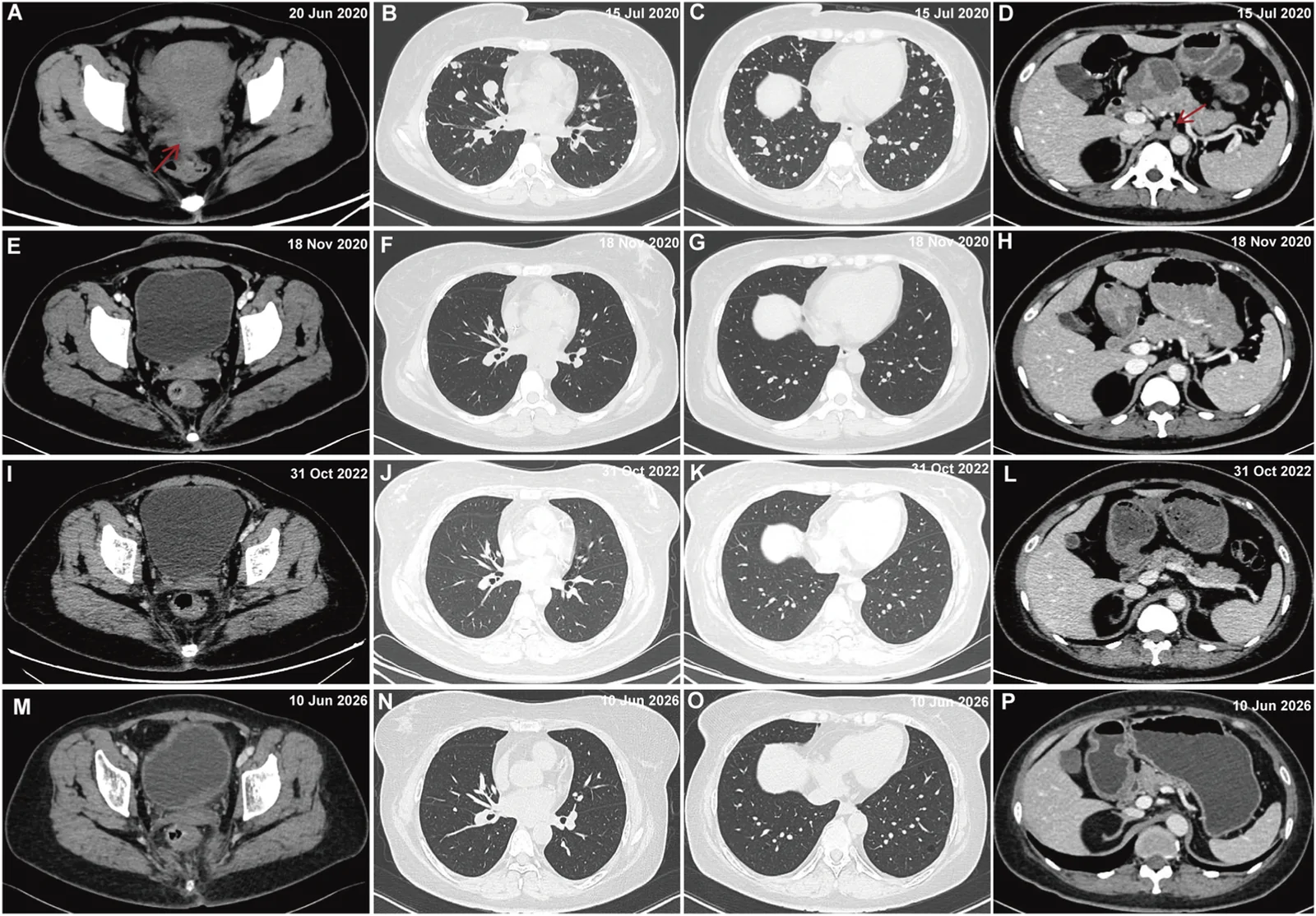
Serial CT imaging demonstrating durable complete tumor remission. Baseline CT shows primary cervical tumor and multiple metastatic lesions **(A–D)**. All lesions completely disappeared after induction therapy, meeting complete response criteria defined by RECIST version 1.1 **(E–H)**. Sustained complete remission is observed during initial tislelizumab maintenance therapy **(I–L)**. Persistent complete response was maintained until June 2026 after safe tislelizumab rechallenge **(M–P)**.

After multidisciplinary team (MDT) discussion and shared clinical decision-making with the patient, six cycles of induction therapy were administered from July 16 to 29 October 2020: nab-paclitaxel 400 mg on day 1, cisplatin 120 mg on day 2, bevacizumab 500 mg on day 1, and tislelizumab 200 mg on day 1. Tislelizumab, a domestic programmed death-1 (PD-1) inhibitor, was selected owing to economic considerations. A partial response (PR) was achieved after two cycles, with continuous tumor regression observed following four cycles. Repeat PET-CT performed on 20 November 2020 showed no abnormally hypermetabolic lesions throughout the body (Figure 1C). Contrast-enhanced CT demonstrated no residual lesions of the primary tumor or distant metastatic lesions (Figures 2E–H). The treatment response was evaluated as CR per Response Evaluation Criteria in Solid Tumors (RECIST) version 1.1.

Four cycles of combination maintenance therapy (nab-paclitaxel 400 mg day 1 plus tislelizumab 200 mg day 1) were delivered from 21 November 2020 to 27 January 2021. Single-agent tislelizumab maintenance (200 mg once every 3 weeks) was initiated on 24 February 2021, concurrent with pelvic intensity-modulated radiotherapy combined with intracavitary brachytherapy administered between February 26 and 23 April 2021. Regular follow-up visits were conducted from June 2021 to January 2023; serial imaging consistently demonstrated persistent CR (Figures 2I–L), the patient maintained an Eastern Cooperative Oncology Group (ECOG) performance status of 0, and no remarkable adverse events were documented.

In January 2023, approximately 23 months after initiation of single-agent tislelizumab maintenance, the patient developed progressive dyspnea without chest pain or chest tightness. Emergency laboratory testing demonstrated drastically elevated high-sensitivity cardiac troponin I (hs-cTnI) at 25,418.9 pg/mL (reference range <34.2 pg/mL). Creatine kinase-MB (CK-MB) was 40.44 ng/mL, creatine kinase (CK) 830 U/L, lactate dehydrogenase (LDH) 518 U/L, lactate dehydrogenase-1 (LDH-1) 108 U/L, and mitochondrial aspartate aminotransferase (mAST) 23 U/L; these markers collectively indicated acute myocardial necrosis. Elevated B-type natriuretic peptide (BNP) at 246 pg/mL suggested impaired cardiac function. Electrocardiography showed nonspecific T-wave inversion, while transthoracic echocardiography revealed a left ventricular ejection fraction (LVEF) of 61% without regional wall motion abnormalities. Coronary angiography performed on 2 February 2023 demonstrated 90% stenosis in the proximal left anterior descending artery, 70%–80% stenosis in the distal left circumflex artery (Figures 3A,B), and plaque infiltration within the right coronary artery, suggesting a diagnosis of NSTEMI. Percutaneous coronary intervention (PCI) was performed, comprising drug-eluting stent implantation in the left anterior descending artery and drug-coated balloon angioplasty of the circumflex artery (Figures 3C,D). Following PCI, dual antiplatelet therapy was initiated: aspirin 0.1 g once daily combined with ticagrelor 90 mg twice daily. Rosuvastatin 10 mg was administered at bedtime for lipid regulation; sustained-release metoprolol succinate 47.5 mg once daily and isosorbide dinitrate tablets 5 mg three times daily were given for cardioprotection. Glycemic control was maintained with glimepiride 2 mg once daily, pioglitazone 30 mg once daily, and linagliptin 5 mg once daily. Upon completion of the dual antiplatelet treatment course, ticagrelor was discontinued, and aspirin monotherapy was retained long-term. The patient developed severe COVID-19-associated pneumonia postoperatively and was discharged on 8 February 2023 after targeted supportive treatment.

**FIGURE 3:**
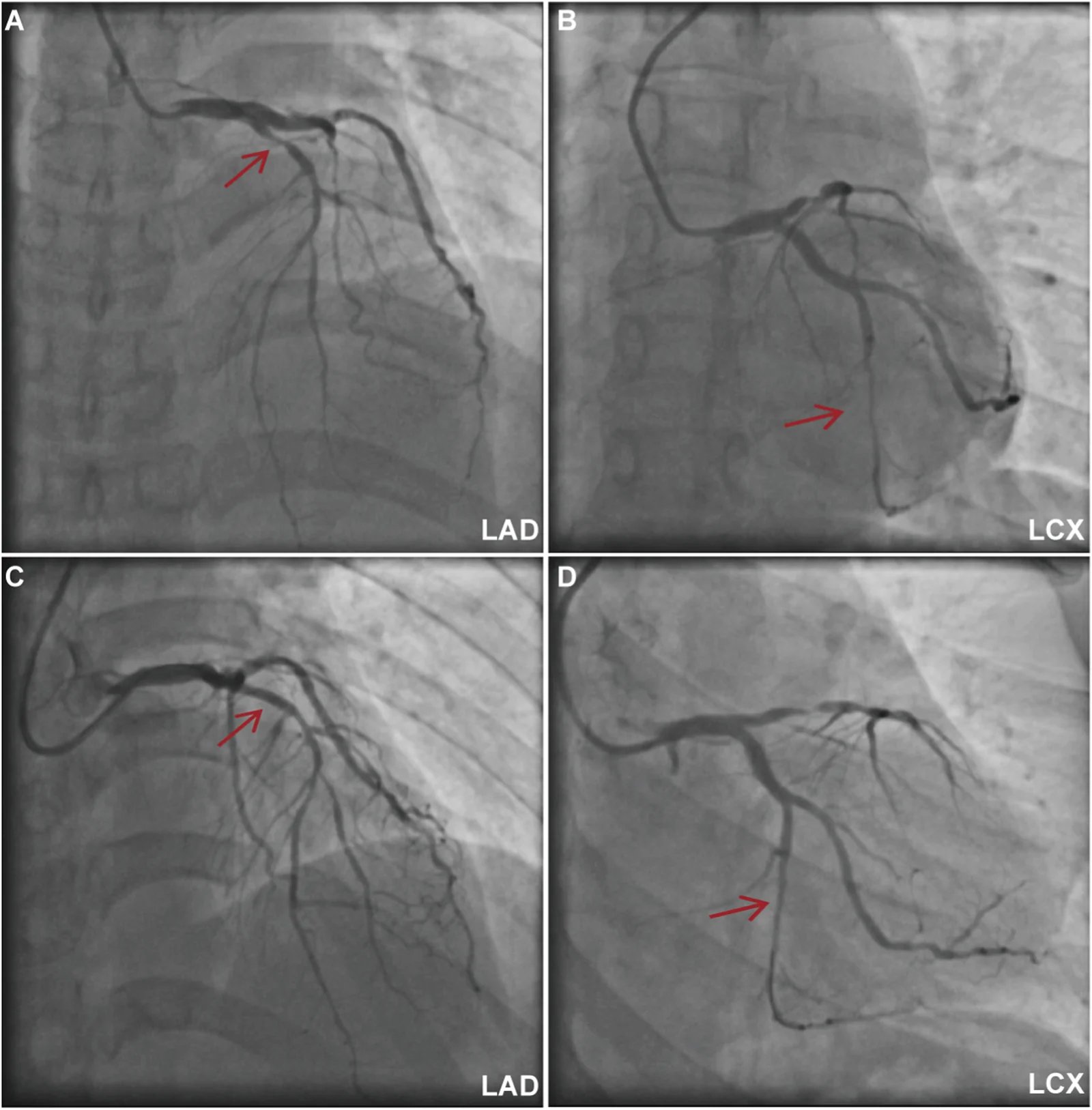
Coronary angiography images before and after percutaneous coronary intervention (PCI) in a patient with immune checkpoint inhibitor-associated non-ST-segment elevation myocardial infarction. Severe 90% stenosis of the proximal left anterior descending artery (LAD) **(A)**. 70%–80% stenosis of the distal left circumflex artery (LCX) **(B)**. Post-PCI angiogram of the proximal LAD lesion following drug-eluting stent implantation **(C)**. Post-PCI angiogram of the distal LCX lesion after drug-coated balloon angioplasty **(D)**.

Comprehensive multidisciplinary reassessment involving oncologists, cardiologists and endocrinologists was conducted after normalization of hs-cTnI, stable LVEF and resolution of active infection. Single-agent tislelizumab maintenance was safely reinitiated on 13 March 2023 and continued until August 2024. After discontinuing tislelizumab in August 2024, the patient’s cardiovascular maintenance regimen consisted of dual antiplatelet therapy (aspirin 0.1 g once daily plus ticagrelor 90 mg twice daily), rosuvastatin 10 mg once daily to stabilize vascular plaques, sustained-release metoprolol succinate 47.5 mg once daily, and amlodipine besylate 10 mg once daily for blood pressure control; during an outpatient follow-up in April 2025, poor blood pressure control was observed, so valsartan capsules 80 mg once daily were added to the original regimen and ticagrelor was discontinued, and the medications administered at the last follow-up in June 2026 were as follows: aspirin 0.1 g once daily, rosuvastatin 10 mg once daily, amlodipine besylate 10 mg once daily, valsartan 80 mg once daily, and sustained-release metoprolol succinate 47.5 mg once daily. After treatment resumption, follow-up assessments were performed every 3–6 months. Persistent CR was maintained until June 2026 (Figures 2M–P). No recurrent cardiovascular events were documented. The patient remained in New York Heart Association (NYHA) class I, with normal myocardial enzyme levels, electrocardiogram, and echocardiography. No additional severe immune-related adverse events (irAEs) were observed. At the last follow-up (June 2026), overall survival (OS) exceeded 71 months, and duration of response (DOR) exceeded 67 months. The timeline of the patient’s treatment course is illustrated in Figure 4.

**FIGURE 4:**
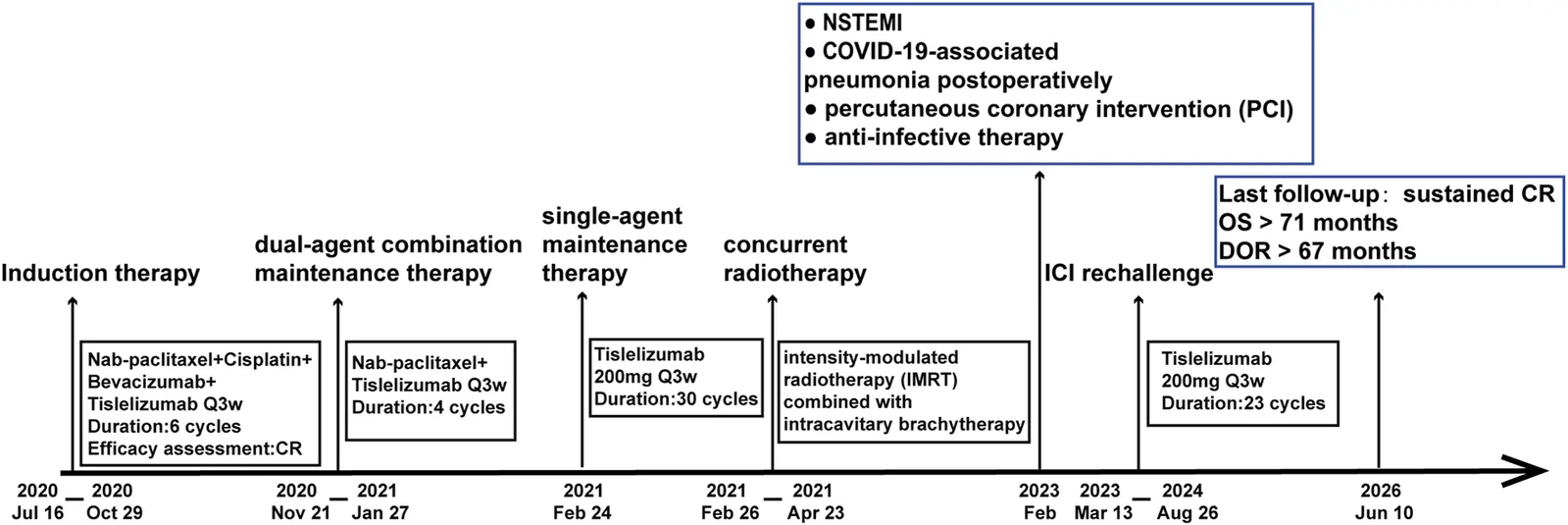
Timeline of treatment, immune-related adverse event and long-term clinical outcomes. The patient received induction chemotherapy combined with bevacizumab and tislelizumab, followed by maintenance immunotherapy plus pelvic radiotherapy, and achieved persistent complete response. In February 2023, the patient developed ICI-related non-ST-segment elevation myocardial infarction (NSTEMI) and received emergency percutaneous coronary intervention (PCI). After multidisciplinary team assessment, tislelizumab was safely reinitiated, and sustained disease remission lasted until June 2026.

## 3 Discussion

The patient in this case suffered from FIGO stage IVB cervical squamous cell carcinoma with a PD-L1 CPS of 65, rendering PD-L1 a core actionable therapeutic target. With the synergistic effects of chemotherapy, anti-angiogenic therapy and radiotherapy, tislelizumab remodeled anti-tumor immunity and induced immune memory. Even though the tumor presented an immunosuppressive immune-cold phenotype inherently common in pMMR cervical cancers, the patient still achieved deep and durable remission. This case also suggests that MMR/MSI status alone cannot fully predict the clinical benefits of ICIs, and pMMR status is not equivalent to an “immune-cold phenotype”. As illustrated by the advanced prostate cancer case reported by Higa et al., treatment eligibility for advanced tumors should not rely on a single target or biomarker. In this patient, prior single-gene *BRCA* testing was negative and failed to rule out Lynch-syndrome-associated alterations. Comprehensive FoundationOne profiling subsequently detected MSI-H, a *BRCA2* mutation, and an *MSH2* exon deletion, confirming Lynch syndrome and guiding ICI initiation (Higa et al., 2025). At present, the crosstalk mechanisms within the tumor immune microenvironment are becoming increasingly complex, and the continuous discovery of novel immune targets and biomarkers has promoted the optimization of therapeutic strategies. For example, lymphocyte-activation gene 3 (LAG-3), T cell immunoglobulin and mucin-domain containing-3 (TIM-3), and T cell immunoreceptor with Ig and ITIM domains (TIGIT) are highly expressed on exhausted T cells in the tumor immune microenvironment. Combined blockade of PD-1 and the above novel checkpoints can enhance T cell responses to tumor antigens, serving as a highly promising combination therapeutic strategy. TIGIT participates in suppressing the anti-tumor functions of natural killer (NK) cells and CD8^+^ T cells, while antibodies targeting TIM-3 and TIGIT are still under clinical exploration for solid tumors (Narote et al., 2025). For this patient with pMMR advanced cervical cancer, the above novel immune targets provide potential combination therapeutic options if disease progression occurs in the future.

In 2020, when the patient initiated treatment, no clinical guidelines recommended ICIs as first-line therapy for advanced cervical cancer; only pembrolizumab was approved for second-line treatment of PD-L1-positive patients. At that time, tislelizumab, an IgG4 anti-PD-1 monoclonal antibody, had been investigated in hematological malignancies and various advanced solid tumors but had not yet received approval for cervical cancer indications (Wendel and Leath, 2020; Lang et al., 2025). Considering the patient’s limited financial resources and the proven survival benefits together with the manageable safety profile of tislelizumab across multiple solid tumors (Nguyen et al., 2025), off-label individualized experimental treatment with tislelizumab was administered after institutional ethical review and comprehensive informed consent. Subsequent data from clinical trial NCT05247619 demonstrated that the combination of tislelizumab, low-dose bevacizumab and platinum-based chemotherapy yielded an ORR of 91.5% as first-line therapy for recurrent/metastatic cervical cancer (Wen et al., 2025), findings highly consistent with the clinical benefit observed in this case.

The patient developed AMI after 23 months of maintenance monotherapy with tislelizumab. Emergency testing revealed markedly elevated cardiac injury biomarkers, including hs-cTnI, CK-MB, LDH-1, mAST, and BNP. Echocardiography demonstrated an LVEF of 61% without regional wall motion abnormalities, and serial electrocardiograms showed no extensive dynamic ST-T changes or refractory arrhythmias. Coronary angiography identified 90% stenosis in the proximal left anterior descending artery and 70%–80% stenosis in the distal circumflex artery, strongly indicating organic obstructive coronary artery lesions. Cardiac magnetic resonance (CMR) and endomyocardial biopsy (EMB) were not performed due to the patient’s urgent clinical status. With respect to differential diagnosis, the clinical manifestations in this case were more consistent with NSTEMI complicated by ICI-accelerated atherosclerosis than with overt immune-mediated myocarditis. Two key lines of evidence support this conclusion. First, coronary angiography revealed severe multivessel stenosis, whereas ICI-associated myocarditis is typically unaccompanied by significant organic coronary obstruction. Second, echocardiography showed no abrupt reduction in LVEF or diffuse wall motion abnormalities. Notably, the patient’s symptoms resolved rapidly following PCI, and neither glucocorticoids nor immunosuppressive agents were required post-procedure. Cardiac function remained stable over more than 3 years of follow-up (NYHA class I), with no worsening heart failure, arrhythmias, or recurrent elevation of myocardial injury markers. This clinical course is distinctly inconsistent with the typical features of ICI myocarditis, which is characterized by rapid deterioration, corticosteroid dependence, and high recurrence risk. Mechanistically, the PD-1/PD-L1 pathway acts as a negative regulator of atherosclerosis. Blockade of this pathway by ICIs activates pro-atherogenic T cell responses, increases inflammatory cell infiltration within atherosclerotic plaques, and triggers plaque rupture (Drobni et al., 2020; Mulholland et al., 2025). Drobni et al. reported that ICI exposure confers a three-to fivefold increased risk of cardiovascular events (adjusted HR = 3.3, 95% CI 2.0–5.5), with excess risk predominantly observed within the first 2 years after treatment initiation (Drobni et al., 2020), a finding highly consistent with our patient’s clinical timeline.

In addition to the aforementioned cardiovascular toxicities of ICIs, accelerated progression of coronary atherosclerotic lesions in the present patient may stem from additive proatherogenic insults conferred by sequential chemotherapy and anti-VEGF treatment. By blunting VEGF signaling, bevacizumab impairs endothelium-dependent vasorelaxation mediated by nitric oxide (NO) and prostacyclin (PGI_2_), thereby increasing the risks of hypertension and arterial thromboembolism. Pooled meta-analyses reveal an approximately two-fold higher risk of arterial thromboembolic events among patients receiving bevacizumab (95% CI, 1.05–3.75) (Li and Kroetz, 2018; Scappaticci et al., 2007). Similarly, cisplatin exerts direct cytotoxic damage to endothelial cells and triggers endothelial apoptosis, upregulates thromboxane release, facilitates platelet activation and aggregation, and stimulates procoagulant microparticle shedding. These cascades collectively create a systemic hypercoagulable state and heighten long-term cardiovascular risk (Herrmann et al., 2016). The 27-month latency from the last bevacizumab infusion to AMI onset precludes classification of this event as an acute, immediate bevacizumab-associated adverse effect. Instead, this AMI constitutes an atherosclerotic cardiovascular event shaped by a composite burden of conventional vascular risk factors and cumulative antineoplastic exposure. Discontinuation of bevacizumab fails to resolve residual cardiovascular risk; progressive plaque deposition and sustained elevation in myocardial infarction risk persist during subsequent ICI maintenance therapy. For patients with malignancy complicated by metabolic vasculopathy who received combination bevacizumab–ICI regimens, standardized cardio-oncology pathways mandate long-term surveillance encompassing blood pressure, lipid profiles, glycemic status, and coronary plaque/calcification load. Intensified secondary prevention measures should be implemented per validated ASCVD risk stratification frameworks.

With the rapid advancement of cardio-oncology research, accumulating evidence indicates that anticancer drug-related cardiovascular adverse events are not solely attributable to pharmaceutical agents; host molecular profiles and genetic predisposition also exert critical influences. In a pair of sisters with ovarian cancer reported by Liu et al., in 2025, both harbored a novel missense variant, *TFAM* c.98 T>A. While this variant alone does not cause cardiomyopathy, it specifically predisposes patients to heart failure upon niraparib administration (Liu et al., 2025). This study implies that genetic susceptibility may modify drug-induced cardiotoxicity: patients carrying specific germline variants can develop drastically divergent cardiac outcomes even with equivalent drug exposure. In the context of the present case, the patient had conventional cardiovascular risk factors, including diabetes mellitus and hypertension, yet her coronary lesions progressed rapidly to severe multivessel stenosis within just 2 years of ICI therapy. This rate of progression far exceeds expectations based on the natural history of conventional risk factors alone. Therefore, beyond classic cardiovascular comorbidities, unidentified molecular or genetic susceptibility factors may amplify the proatherogenic effects of ICIs.

Notably, several limitations of the present case deserve emphasis. First, baseline coronary imaging prior to anticancer treatment was unavailable, precluding direct visualization of dynamic changes in plaque burden before and after ICI administration. Second, severe coronary stenosis and ICI-associated myocarditis are not mutually exclusive diagnoses. Although the patient maintained long-term cardiac stability without glucocorticoid therapy following PCI and experienced no recurrent cardiac events over more than 3 years of follow-up—a clinical trajectory inconsistent with the typical course of ICI myocarditis—CMR and EMB were not performed during the AMI episode. Consequently, subclinical ICI-related myocardial injury cannot be entirely ruled out. Third, the patient had concomitant diabetes mellitus and hypertension, both independent risk factors for ASCVD. It is therefore impossible to fully disentangle the superimposed effects of progressive underlying cardiovascular comorbidities and ICI-mediated proatherogenic injury. A definitive causal relationship between tislelizumab and this episode of NSTEMI cannot be established, and subclinical ICI-associated myocardial injury remains a differential consideration. Fourth, germline genetic testing and endomyocardial biopsy were omitted due to the patient’s critical acute presentation, leaving unresolved whether the patient harbored pathogenic variants in genes regulating immune checkpoint signaling or mitochondrial function (e.g., TFAM), or atherosclerosis susceptibility loci. The above points constitute the key limitations of this case report. For patients scheduled for prolonged ICI therapy with concurrent cardiometabolic risk factors, baseline evaluation via coronary artery calcium scoring or coronary computed tomography angiography (CTA) is recommended prior to treatment initiation. If elevated myocardial injury biomarkers emerge during ICI maintenance, timely CMR should be performed to differentiate myocarditis from ischemic myocardial damage. Furthermore, targeted germline sequencing may be conducted in future analogous cases complicated by ICI-triggered acute coronary syndromes, to explore potential correlations between specific germline variants and ICI-accelerated atherosclerotic progression.

Restarting ICIs after severe irAEs represents a major clinical challenge. To date, no standardized guidelines have been established, and clinical decisions rely primarily on individualized comprehensive risk assessment. The present patient had FIGO stage IVB cervical cancer with a high baseline tumor burden and an extremely high risk of disease recurrence. Following a cardiovascular adverse event and complete coronary revascularization, rigorous MDT evaluation combined with the patient’s treatment preference supported the reintroduction of tislelizumab monotherapy for maintenance under an acceptable risk-benefit profile. Several critical prerequisites ensured safe reinitiation: complete resolution of obstructive coronary lesions via PCI, full normalization of myocardial injury biomarkers, sustained left ventricular ejection fraction above 60%, and exclusion of active systemic infection. At present, high-level evidence defining the optimal duration of ICI maintenance therapy for advanced cervical cancer remains lacking. The 2-year treatment cap set in pivotal trials such as KEYNOTE-826 merely serves as a pre-specified drug exposure control criterion for trial design, rather than an optimal discontinuation endpoint validated by randomized controlled trials (Monk et al., 2023). Current guidelines do not mandate fixed 2-year treatment cessation; instead, they recommend continuous immunotherapy until disease progression or intolerable toxicity occurs. Retrospective cohort studies have demonstrated that some patients who achieve CR experience disease relapse after ICI discontinuation; however, no robust clinical biomarkers are currently available to identify patients eligible for safe treatment cessation (Marron et al., 2021). The patient achieved first complete remission in November 2020 and maintained deep, durable tumor regression for 45 months until treatment discontinuation in August 2024, with favorable overall tolerance. Extended maintenance with tislelizumab effectively consolidated anti-tumor immune responses. After a treatment duration substantially exceeding the conventional 2-year clinical course, the patient’s anti-tumor benefit reached a plateau, and incremental gains from continued therapy gradually diminished. Considering the patient’s long-term ICI exposure and persistent cumulative cardiovascular risk post-PCI, dynamic risk-benefit re-evaluation by the MDT led to timely discontinuation of tislelizumab in August 2024. The patient sustained complete remission for 17 months after treatment reinitiation, and during 22 months of follow-up after treatment cessation, the patient exhibited no tumor recurrence, no recurrent cardiovascular adverse events, and stable cardiac function. These findings support the safety and feasibility of this individualized strategy consisting of ICI reintroduction, prolonged maintenance, and elective discontinuation. In conclusion, prospective controlled data comparing fixed-duration ICI therapy versus individualized prolonged maintenance are still lacking. The >2-year immunotherapy maintenance administered in this case reflects an individualized clinical decision rather than a standardized regimen. Large-scale randomized controlled trials are warranted to clarify the optimal maintenance duration and precise timing for ICI discontinuation in patients with advanced cervical cancer.

With the widespread application of ICIs in earlier disease stages and long-duration maintenance regimens, cardio-oncology collaborative management should be established as standard clinical practice, including ASCVD risk stratification before treatment, germline genetic testing prior to treatment initiation, heightened vigilance for atypical ischemic symptoms during therapy, routine monitoring of cardiac troponin and electrocardiogram, and long-term cardiovascular follow-up (Lyon et al., 2022). For patients with cardiovascular risk factors, baseline coronary evaluation (such as coronary calcium score or coronary CTA) facilitates identification of high-risk populations and guides individualized monitoring strategies.

## 4 Conclusion

This case demonstrates that tislelizumab-based chemoimmunotherapy can induce durable complete remission exceeding 5 years in patients with FIGO stage IVB pMMR cervical cancer complicated by metabolic disorders. In addition, this case indicates that ICIs may correlate with accelerated atherosclerotic cardiovascular events and supports the feasibility of safe immunotherapy reinitiation following myocardial infarction via MDT assessment. Given the expanding indications and prolonged treatment cycles of ICIs, pretreatment germline genetic testing and cardiovascular risk stratification help identify patients at high risk of cardiotoxicity. Patients receiving ICI therapy should undergo comprehensive cardiovascular risk assessment and optimized prophylactic pharmacotherapy, followed by close surveillance. In clinical practice, a comprehensive collaborative cardio-oncology management model throughout treatment should be implemented to optimize the balance between the anti-tumor efficacy of immunotherapy and its cardiovascular safety risks.

## Data Availability

The original contributions presented in the study are included in the article/supplementary material, further inquiries can be directed to the corresponding author.
